# Effects of visual diet on colour discrimination and preference

**DOI:** 10.1098/rspb.2024.0909

**Published:** 2024-09-18

**Authors:** Alice E. Skelton, John Maule, Simeon Floyd, Beata Wozniak, Asifa Majid, Jenny M. Bosten, Anna Franklin

**Affiliations:** ^1^ The Sussex Colour Group & Baby Lab, School of Psychology, University of Sussex, Brighton BN1 9RH, UK; ^2^ Nature & Development Lab, School of Psychology, University of Sussex, Brighton BN1 9RH, UK; ^3^ Statistical Perception Lab, School of Psychology, University of Sussex, Brighton BN1 9RH, UK; ^4^ Colegio de Ciencias Sociales y Humanidades, Universidad San Francisco de Quito, Quito, Ecuador; ^5^ Department of Experimental Psychology, University of Oxford, Oxford OX2 6GG, UK; ^6^ Sussex Vision Lab, School of Psychology, University of Sussex, Brighton BN1 9RH, UK

**Keywords:** colour, perception, scene statistics, discrimination, preference

## Abstract

To what extent is perception shaped by low-level statistical regularities of our visual environments and on what time scales? We characterized the chromatic ‘visual diets’ of people living in remote rainforest and urban environments, using calibrated head-mounted cameras worn by participants as they went about their daily lives. All environments had chromatic distributions with the most variance along a blue–yellow axis, but the extent of this bias differed across locations. If colour perception is calibrated to the visual environments in which participants are immersed, variation in the extent of the bias in scene statistics should have a corresponding impact on perceptual judgements. To test this, we measured colour discrimination and preferences for distributions of colour for people living in different environments. Group differences in the extent of blue–yellow bias in colour discrimination were consistent with perceptual learning in local environments. Preferences for colour distributions aligned with scene statistics, but not specifically to local environments, and one group preferred distributions along an unnatural colour axis orthogonal to that dominant in natural scenes. Our study shows the benefits of conducting psychophysics with people at remote locations for understanding the commonalities and diversity in human perception.

## 1. Introduction

It is generally accepted that the environments in which we live influence the structure and function of our sensory and perceptual systems [1]. For example, the efficient coding framework proposes that sensory systems have evolved to encode natural environments efficiently and to adapt to them [2]. In support of the idea that perception is calibrated to perceptual environments, the visual system is aligned with various statistical regularities in natural scenes such as luminance, contrast, colour and spatial structure [3–5]. For example, both visual sensitivity and aesthetic preferences peak for stimuli with a spatial structure (e.g. fractal dimension and spectral slope) in the range that is typical of natural scenes [6–8]. There are multiple timescales on which calibration to natural scenes could occur. Perception could be optimized to the ancestral environment during evolution. In addition, due to plasticity and long-term adaptation [9], perception could also calibrate to the environments that form an individual’s visual diet during their lifetime. If within-lifetime calibration occurs, we expect that differences in statistical regularities across environments should be accompanied by differences in perception: environmental differences should be a source of perceptual diversity [10]. However, there has been little investigation of cross-environmental differences in scene statistics and their impacts on perception. Here, we use colour as a model system to investigate timescales of calibration to natural scenes.

Colour perception is thought to be tuned to natural environments in a number of ways [11,12]. The spectral sensitivity functions of photoreceptors are thought to have evolved to allow us to discriminate fruit from foliage in the diets of our primate ancestors [13,14], or socially relevant cues in the colour of skin [15]. Postreceptorally, the two predominant pathways that carry colour information from the retina to the lateral geniculate nucleus appear to be tuned to decorrelate colour information in natural scenes [16]. There has been recent interest in a further level of alignment between colour perception and the colour statistics of natural scenes. Colour discrimination is poorest along a roughly ‘blue–yellow’ axis intermediate between the colour axes of the two retinogeniculate colour pathways, and visual environments vary most along this intermediate axis [4,17–20]. We refer here to this effect as the ‘blue–yellow bias’ in perception or environment. It has been suggested, in line with an efficient coding framework, that long-term adaptation to contrasts [19] produces a ‘range-accuracy trade-off’, where the larger range of colours along this axis must be represented by the visual system with lower fidelity [4,21,22]. Aesthetic judgements of colour have also been linked to the colour variation in natural environments [7,23]. Juricevic *et al*. [7] found that ratings of ‘artistic merit’ for chromatic Mondrians (abstract multi-coloured patches) are greatest, and visual discomfort lowest, for Mondrians with colours that vary roughly along the blue–yellow axis, and therefore there appears to be a blue–yellow bias in aesthetic judgements as well. Juricevic *et al*. [7] proposed an explanation for their results that also relates to the efficient coding framework, that any stimuli that deviate strongly from the statistics of natural scenes are processed inefficiently and lead to visual discomfort and low aesthetic ratings.

As outlined above, the extent to which visual perception tunes to environmental statistics ontogenetically within an individual’s lifetime is unclear. For colour, the results of two cross-seasonal studies imply that exposure to different chromatic statistics in different seasons can lead to small but measurable differences in aspects of colour appearance and colour discrimination, implying a level of tuning within the lifetime [24,25]. However, neither study quantified the changing colour statistics of the environments to predict directional effects on perception. Experiments on short- and medium-term adaptation also predict that colour perception is malleable to the colour statistics of the experienced environment [20,26]. Although there are correlations between colour perception and natural scene statistics and there are some hints that colour perception might be malleable to environmental influences, we don’t know how immersion in distinct naturally occurring environments affects colour perception. For environments as different from one another as lush rainforests and urban built environments, what are the impacts on colour perception?

We set out to measure the blue–yellow bias in colour scene statistics and its impact on colour discrimination and preferences for populations living in radically different visual environments. Specifically, using colour-calibrated iPads (Apple, Cupertino, CA, USA) in controlled lighting conditions, we measured colour perception in the following three groups: a group from the Chachi population who are indigenous to the Esmeraldas rainforest in Ecuador and who live immersed in a rainforest environment; a group from the Chachi population who have migrated to Ecuador’s capital city, Quito; and a group in Sussex in the Southern UK (see electronic supplementary material, ‘Information about the Chachi population’). A rainforest environment was chosen as it has previously been shown that the blue–yellow bias is reduced in lush environments relative to arid ones [27]. The Chachi living in the Esmeraldas have relatively limited exposure to artificial light sources and industrialized objects, meaning that their exposure to the rainforest environment should be a large part of their visual diets. Sampling the Chachi population who have migrated to Quito enabled us to dissociate environmental from genetic influences on perception. Sussex was included as an additional urban environment that is at a different latitude from Quito.

Using calibrated head-mounted cameras, we captured, from participants’ perspectives, the chromatic scene statistics of a random sample of scenes encountered in their daily lives. Our design allows us to arbitrate between different possibilities. If colour perception is tuned to the colour statistics of the contemporary local environment, differences in colour statistics would be mirrored by differences in colour perception. If the environments differ significantly in their colour statistics but perception does not differ accordingly, either long-term genetic tuning or no causal relationship is implied. We found significant differences in colour statistics between environments and significant differences in perception between groups, but the effects we found were not as predicted under the efficient coding framework. Rather, our results imply that group differences in colour discrimination could be driven by perceptual learning and that an account of preferences via efficient coding must be culturally moderated. Our study provides a blueprint for conducting psychophysics with populations in remote locations who are not ‘WEIRD’ (Western, educated, industrialized, rich, democratic), which must be included in cognitive science for a complete understanding of commonalities and diversity in human perception.

## 2. Methods

### (a) Participants

Ecuadorian participants were all members of the indigenous Chachi community living in the Esmeraldas rainforest in northern Ecuador and working in agriculture or fishing, or living in the capital Quito and working in hospitality or construction. For both Ecuadorian groups, the highest level of education was high school, and participants spoke both Cha’palaa and Spanish (see electronic supplementary material, ‘Information about the Chachi population’). British participants were mainly students at the University of Sussex and lived in the seaside city of Brighton. A total of 175 participants took part in the behavioural tasks (table 1), and 25 participants took part in the image acquisition (Esmeraldas *n* = 7, age *M* = 26.9, s.d. = 3.2, four females and three males; Quito *n* = 12, age *M* = 25.3, s.d. = 4.5, five females and seven males; Sussex *n* = 6, age *M* = 33.7, s.d. = 3, four females and two males). Participants were not screened for colour vision deficiency (CVD) using standard diagnostic tests, but we analysed our colour discrimination data for CVD (see §3).

**Table 1.** Participant numbers and demographics for the behavioural tasks.

task	location	total *n*	*n* included	mean age (years)	s.d. age (years)	male : female
discrimination thresholds						
	Esmeraldas	31	31^a^	23.71	5.06	20 : 11
	Quito	37	36^a^	24.97	4.40	18 : 18
	Sussex	38	38^a^	26.12	4.05	18 : 19 (1 NB)
preference rating						
	Esmeraldas	31	29^a^	23.5	4.9	15 : 14
	Sussex	50	50	27.8	6.1	26 : 23 (1 NB)
preference ranking						
	Quito	37	24^a^	25.5	4.3	10 : 14
	Sussex	19	19	24.3	3.4	3 : 16

^a^ Mean age, s.d. age and male : female ratios are for included participants only.

NB, non-binary gender.

### (b) Perceptual tasks

There were two perceptual tasks to measure colour discrimination and colour preferences. The colour discrimination task asked participants to select colourful targets among grey distractors. The colour preference task asked participants to rate their preferences for coloured patterns. Participants took part individually within a darkened lab room or blackout tent (Sussex) or inside a blackout tent situated inside a building/dwelling (Quito and Esmeraldas). Tasks were conducted at a comfortable viewing distance for using a tablet display, so the sizes of stimuli were not precisely controlled in degrees of visual angle. The experimenter explained the task verbally in the preferred local language (English in Sussex, Spanish/Cha’palaa in Quito and Esmeraldas), and then the participant began the tasks on the iPad tablet, while the researcher provided verbal support for participants to understand the task where needed. Ecuadorian participants completed both tasks and always completed the colour discrimination task first, and UK participants completed one task only. In order to present calibrated coloured stimuli, iPads were gamma corrected and RGB to LMS conversion matrices were calculated using measurements acquired with a PR655 SpectraScan photoradiometer (PhotoResearch, Chatsworth, CA), following the method described in Tang *et al*. [28]. Calibrations were conducted separately for each specific iPad unit used in the study (see electronic supplementary material, ‘iPad calibration’ for the gamma curves and spectra).

#### (i) Colour discrimination task

**Figure 1.**
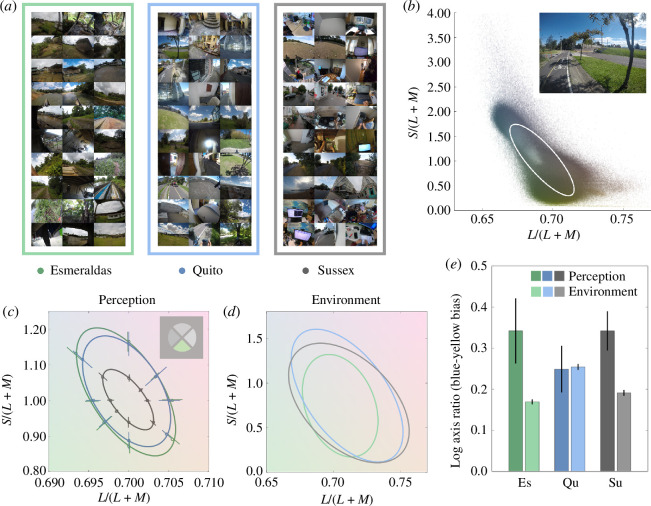
Scene statistics and colour discrimination are blue–yellow biased, but the group whose colour discrimination is least blue–yellow biased are from the most blue–yellow biased environment. (*a*) Montages of images gathered at Esmeraldas (green frame), Quito (blue frame) and Sussex (grey frame). (*b*) The colour distribution of pixels in an example image from Quito (inset). The chromaticity of each pixel is plotted in a version of the MacLeod–Boynton chromaticity diagram [29]. To the distribution of chromaticities in each image we fit a s.d. ellipse. (*c*) Mean colour discrimination ellipses for participants at each location. Error bars are 95% confidence intervals. Inset is a diagram of the 4AFC stimulus. The mean discrimination ellipses for participants at all three locations are elongated along the negative diagonal of the MacLeod–Boynton chromaticity diagram [29], suggesting colour discrimination is poorer along the roughly blue–yellow axis than along the orthogonal green–magenta axis. (*d*) s.d. ellipses fit to the distributions of chromaticities in the images acquired at each location. Fits plotted are to a random sample of pixels from all images from each location (10 000 pixels from each individual image). s.d. ellipses for all three locations are biased along the negative diagonal of the MacLeod–Boynton chromaticity diagram [29], meaning that there is more chromatic variance in natural scenes along the roughly blue–yellow axis than along the orthogonal magenta–green axis. (*e*) Mean log axis ratios of the distributions of colours in natural environments at each of the three locations (light bars) and mean log axis ratios of colour discrimination ellipses for participants at each of the three locations (dark bars). Error bars are 95% confidence intervals. Axis ratios are calculated following a transformation of the s.d. ellipses (e.g. panel (*b*)) to equate variances along the *x*- and *y*-axes. The axis ratio is defined as the ratio of the length of the ellipse axis oriented along the negative diagonal to the length of the ellipse axis oriented along the positive diagonal. If images are ‘blue–yellow biased’ (i.e. contain more chromatic variance along the negative than positive diagonal) then the log axis ratio is greater than 0. Axis ratios are larger than 0 for discrimination ellipses for participants at all three locations and for the s.d. ellipses fit to distributions of colours in natural scenes at all three locations. Both scene statistics and colour discrimination are biased along the blue–yellow axis. However, discrimination ellipses are least blue–yellow biased for participants in Quito, while natural scenes are most blue–yellow biased in Quito.

The colour discrimination task was a custom iPad app programmed in Unity (San Francisco, CA, USA). On each trial, participants saw an annulus divided into four quadrants (see inset in figure 1*c*), where one quadrant was the chromatic ‘target’ and the remaining three quadrants were achromatic distractors metameric with equal energy white that had luminance jitter ± 10% of the mean target luminance of 30 cd m^−2^. The outer radius of the annulus was 1.75 cm (approx. 1.8°), and the inner radius was 0.35 cm (approx. 0.35°). The annulus was presented on a black background. We measured colour discrimination along eight hue axes (figure 1*c*) in a version of the MacLeod–Boynton chromaticity diagram [29] constructed using the Stockman, MacLeod & Johnson [30] cone fundamentals, centred on equal energy white. This chromaticity diagram provides a physiologically relevant representation of colour, where the two ‘cardinal’ axes, *S*/(*L* + *M*) and *L*/(*L* + *M*), represent the activities of the two cone-opponent retinogeniculate colour pathways. Colours varied along eight radial axes, which were increments and decrements along the ‘cardinal’ axes of the chromaticity diagram (*L*/(*L* + *M*) and *S*/(*L* + *M*)), increments and decrements along the ‘blue–yellow’ axis running from unique blue at 476 nm to unique yellow 576 nm [31] and increments and decrements along the reflection of the blue–yellow line through a line parallel to the *S*/(*L* + *M*) axis passing through equal energy white (figure 1*c*).

Participants were asked in their preferred language to find the ‘colourful one’, and first had three highly salient practice trials. On each trial of the testing phase, participants had to tap the coloured target. If a participant correctly selected the target, a 2 s animation and sound were activated. The saturation of the targets varied along the eight axes in the MacLeod–Boynton chromaticity diagram [29]. On each trial, the target had one of the eight possible hues with its saturation determined via eight randomly interleaved staircases, one for each colour axis. For each staircase, targets were initially shown at the highest saturation level, and if the chromatic target was chosen, then the saturation of the target along that hue axis was decreased by a factor of 0.5 on its next presentation. An incorrect response resulted in an increase in saturation by a factor of 1.5 for its next presentation. There were two consecutive staircases for each hue, each ending after 18 reversals.

#### (ii) Preference task

**Figure 2.**
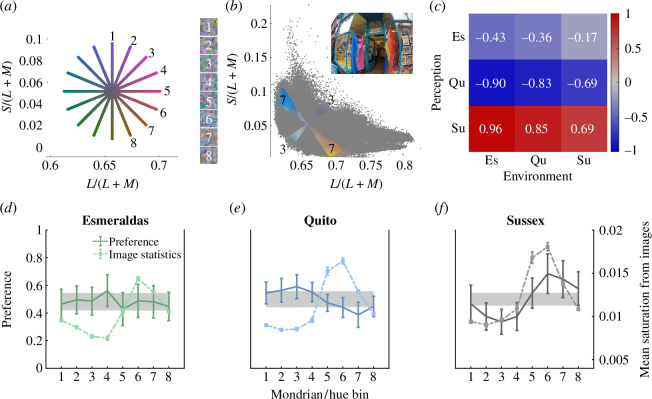
Colour preferences and mean saturation of the environment are related to each other, but not in a location-specific way. (*a*) The eight colour axes in the Macleod–Boynton chromaticity diagram [29] along which Mondrian colours were selected. Axes are straight lines, numbered at one end and spanning the central grey point (Illuminant C). Each axis therefore contains two complementary hues of varying contrasts from grey. Example Mondrians corresponding to each numbered axis (hue angles: 1, 0.0°, 2, 22.5°; 3, 45.0°; 4, 67.5°; 5, 90.0°; 6, 112.5°; 7, 135.0°; 8, 157.5°) are shown to the right. (*b*) The distribution of chromaticities in an example image shown inset to the upper right. Each grey datapoint shows the chromaticity of a pixel from the image. Coloured pixels illustrate the selection of complementary wedges corresponding to the third and seventh Mondrian axes. (*c*) Correlations between the group-level mean preferences for the eight Mondrians for participants in each location and corresponding mean saturations from images captured in each location. Spearman’s correlations show a strong relationship between preferences and visual diet, although not location-specific, for Sussex and Quito participants, while Esmeraldas participants’ preferences have a weak relationship with the statistics of the visual diet in all locations. (*d*–*f*) Mean preferences for participants (solid lines) from Sussex (*n* = 19), Quito (*n* = 24) and Esmeraldas (*n* = 29), and the image statistics (mean saturation across all images captured) from the visual diets at the local environment (dashed lines) (i.e. Esmeraldas images in panel (*d*), Quito images in panel (*e*), Sussex images in panel (*f*)). These data are plotted as a function of the Mondrian axis, numbered as in panel (*a*). Error bars indicate 95% confidence intervals of the mean, both for perception and for environment. The grey shaded areas indicate 95% confidence intervals from 10 000 permutations of the preference data, illustrating the null distribution for preferences given random responding.

For the preference task, stimuli were chromatic ‘Mondrian’ patterns, chosen because prior research has used such stimuli to reveal a blue–yellow bias in the colour preferences of adults in the USA [7]. The Mondrian stimuli were computer generated by placing rectangular elements at random positions over a large ‘canvas’, from which a smaller section was extracted as a stimulus image to avoid edge effects (as in Juricevic *et al*. [7]; see figure 2*a*). Rectangular elements were random in size both horizontally and vertically, with a maximum size of 17% of the final image size and a minimum size of 2% of the final image size, although due to occlusion by elements added later it is possible that even smaller areas were produced. The Mondrian format of the stimuli enabled a distribution of colours to be shown as a single stimulus, and the stimuli also had a spectral slope that approximated that of natural scenes [7]. Mondrians contained colour variation along one of eight axes in the MacLeod–Boynton chromaticity diagram [29] based on the Smith & Pokorny [32] cone fundamentals, and in which the chromatic axes were scaled as in Juricevic *et al*. [7] to approximately equate chromatic contrast and centred on a white point of illuminant C (see equation 1 in [7]). In this colour space, the polar angle around the white point specifies hue, and eccentricity from the white point specifies saturation or colour intensity. For each rectangular element of a particular Mondrian, the chromaticity was randomly selected from one of eight axes through the colour space. The eight axes all passed through the white point such that in any given Mondrian, two complementary hues (e.g. 45° and 225°) were represented at different levels of saturation (figure 2*a*). The maximum saturation was set to a limit which would allow the most saturated example of any hue to be within the gamut of each of our display devices. Within each Mondrian, the luminance of each rectangular element was jittered by up to 50% above or below the mean luminance (approx. 55 cd m^−2^). Mondrian images were generated for each specific iPad unit, ensuring stimuli were calibrated to variations in the display properties of each iPad.

We developed a bespoke iPad app which had two formats—ranking and rating. The ranking version of the preference task began with an array of all eight Mondrians, randomly arranged in a grid on the display. Each Mondrian was 3.4 cm (approx. 3.9°) in height and width. The participant was instructed to choose the one they liked best on each display. After tapping their favourite, it was removed, and the remaining Mondrians were shuffled and presented again. This continued until there was only one Mondrian remaining (the least preferred). In Sussex, this task was completed on an iPad connected as an external display and touch interface to a PC running MATLAB (MathWorks, Natick, MA, USA) and Psychtoolbox [33,34], which was enabled via use of the Wired XDisplay Agent (Splashtop, Cupertino, CA, USA). For Quito, this task was converted to a Unity app for use on the iPad as a standalone device. The appearance, operation and interface of the task were identical in the Psychtoolbox and Unity versions of the task. Participants in Quito (*n* = 24) completed five trials, and participants in Sussex (*n* = 19) completed ten trials (mean ranks from the first five trials and from the last five trials were highly correlated, *r* = 0.96). Data presented in figure 2 for the Sussex and Quito participants are based on this ranking method. Preference data for the Esmeraldas participants are based on a rating task instead. A second field trip to gather ranking data from this group was prevented by the COVID-19 pandemic. Preference data were analysed for 29 Esmeraldas participants as data from two participants was excluded. These participants gave the minimum rating to every Mondrian, and importantly, they also predominantly gave the minimum rating when preferences for familiar objects and preferences for single colours were probed, indicating a general disengagement with the task. Note that inclusion of data from these participants would not affect any statistical inferences. In the rating task, the Mondrian stimuli were 5.7 cm^2^ and approximately 6.5°, and each Mondrian was presented on its own twice. The participant was asked to give a rating of their visual appreciation for the pattern by one of three rating methods (by giving tokens to indicate how much the image was liked, using a 7-point Likert scale, or using a sliding scale (see electronic supplementary material, ‘Preference rating method’). In all versions of the ranking and rating tasks, Mondrian images were presented on a black background. We also gathered data using the rating app in Sussex with a different group of participants (*n* = 50), but data from the ranking task were used in the main analysis for ease of comparison with results from the Quito group (see electronic supplementary material*,* ‘Comparison of preferences from rating and ranking methods for Sussex participants’). We also gathered ranking data from Quito participants for a set of single colours in order to further understand the nature of their preferences (see electronic supplementary material, ‘Preferences for single colours in Quito participants’).

### (c) Quantifying the chromatic statistics of the environment

Sampling of the visual diet involved acquiring images from a first-person perspective using Hero 5/6 head-mounted, colour-calibrated cameras (GoPro, San Mateo, CA, USA) issued to participants living in each of the three testing locations (see electronic supplementary material, ‘Camera calibration’ for the calibration method). The camera was set to timelapse mode, acquiring an image every 30 s, and was set to store the RAW image files. These image files contain the raw sensor data for each pixel in the image, encoded as 14-bit integers (16 384 levels). This raw sensor data has not been modified by the camera’s internal white balance correction and hence can be used to provide an estimate of the chromaticity for each point in the scenes captured. After the camera was set up by the experimenter, the participant wore the camera until the battery expired and images were no longer being captured—typically around 120–150 min (240–300 images). Image acquisition sessions took place throughout the day—with start times in the morning (around 09.00), afternoon (around 13.00) and evening (around 18.00). During image acquisition, participants were instructed to carry out their usual activities, as far as possible. Participants were advised to cover or remove the camera in locations where their own or others’ privacy could be at risk, for example, in bathrooms or changing rooms. They were also advised to remove the camera if asked to, or if they felt under threat. Participants in Quito were advised to avoid going out into busy public spaces for their own safety. A total of 18 331 images were acquired (Esmeraldas = 4690, Quito = 8250 and Sussex = 5391). Images were acquired during the same season as the other tasks (May–June for Esmeraldas, January–February for Quito and July–August for Sussex). The image analysis we report here is based on a subsample of 938 images from Esmeraldas, 1650 images from Quito and 1078 images from Sussex, which included every fifth image in the sequences acquired. We subsampled the images to reduce computational demands, and we judged that sampling every 30 s gave a finer resolution than needed given the frequency with which participants’ points of view changed. Sampling every fifth image enabled us to capture participants’ points of view every 2.5 min, which we judged would enable both a representative sample of the images and an efficient analysis. It is unlikely that downsampling would introduce any biases, as sampled and rejected images occur close in time. However, to confirm this, we analysed a second sample of the same number of images from each group and found no significant differences in the colour statistics we extracted from them (see electronic supplementary material, ‘Image analysis replication’). For the image analysis, images were filtered to exclude pixels that were very dark (RAW R, G or B values lower than 15), since estimates of chromaticity for these pixels are adversely affected by sensor noise. We also filtered out pixels that approached saturation of the sensor (RAW R, G or B values greater than 15 000), since estimates of chromaticity for these pixels were restricted by the camera gamut. Images were submitted for chromatic analysis separately for each behavioural task, each of which quantified the chromatic statistics of the environment in different ways. The two pathways of chromatic analysis also reflected differences between the theoretical frameworks underlying the rationales for each task, and the different LMS cone fundamentals used to create the stimuli presented during each task. One analysis quantified the extent of the blue–yellow bias in the images. A second analysis quantified the mean chromatic contrast present in each scene along each of the axes that defined the Mondrian stimuli in the preference task (see §3 for further details of both image analyses).

## 3. Results

We analysed performance on the colour discrimination and colour preference tasks for each of the three groups. From the images gathered using the head-mounted cameras, we extracted for each task the chromatic scene statistic we expected performance on that task to be related to. We then conducted analyses to investigate the correspondence between perception and chromatic scene statistics across the three groups.

### (a) Colour discrimination and the blue–yellow bias in natural scene statistics

For each of the eight hue axes, we extracted a colour discrimination threshold, which was defined as the difference in colour saturation from the white point needed to correctly identify a coloured target among three achromatic distractors on 45% of trials. Logistic psychometric functions for each hue were fit using tools available as part of the *modelfree* toolbox [35], and saturation thresholds for each hue were chosen as the level of saturation needed for participants to detect targets on 45% of trials, chosen because a minority of participants had high lapse rates. Data were available for all 31 participants in the Esmeraldas group and all 37 participants in the Quito group, but one participant from Quito was excluded because psychometric functions could not be fit to their data. Data were available for 38 participants in the Sussex group.

In order to summarize each participant’s sensitivity to colour, we fit individual ellipses to each participant’s set of eight colour discrimination thresholds. We then transformed the data into a ‘normalized’ version of the MacLeod–Boynton chromaticity diagram [29], where the variances along each of the two cardinal axes were normalized. For discrimination ellipses in the normalized chromaticity diagram, we quantified the blue–yellow bias in discrimination as the ‘log axis ratio’: the log ratio of the length of the ellipse axis oriented along the negative diagonal to the length of the ellipse axis oriented along the positive diagonal [4]. If colour discrimination is biased along roughly the blue–yellow axis (the negative diagonal in the MacLeod–Boynton chromaticity diagram [29]), then the log axis ratio will be greater than 0. If it is biased in the opposite (roughly magenta–green) direction, the log axis ratio will be smaller than 0. If there is no bias along either diagonal, the log axis ratio will be 0.

To characterize the blue–yellow bias in natural environments at each location, we analysed the set of raw format images gathered using the head-mounted camera (e.g. figure 1*a*; see §2). For each image, we plotted the chromaticity of every pixel in a version of the MacLeod–Boynton chromaticity diagram [29] (e.g. figure 1*b*; see §2). We then fit, to the distribution of chromaticities in each image, a s.d. ellipse. For each image, the major axis of the s.d. ellipse is the axis of maximum chromatic variance. After transforming the s.d. ellipse into the normalized version of the MacLeod–Boynton chromaticity diagram, we extracted log axis ratios analogously as for the discrimination ellipses.

For colour discrimination, we found that log axis ratios were significantly greater than 0 for participants at all three locations (9.0 < *t* < 16.3, *p* < 0.001; figure 1*e*), indicating that colour discrimination is blue–yellow biased in all three groups. We found a significant difference in mean log axis ratio between participants at different locations (*F*(2,102) = 3.66, *p* = 0.029), suggesting a difference in the *extent* of the blue–yellow bias. Post hoc Tukey tests indicated that log axis ratios for the Quito participants were significantly smaller than for the Sussex participants (*p* = 0.046), and tended to be smaller than for the Esmeraldas participants (*p* = 0.068). However, a Bayesian ANOVA found that the effect of location was not sensitive (BF_10_ = 1.69). For the images of the local environments, we found that log axis ratios were significantly greater than 0 for all three locations (53.5 < *t* < 68.3, *p* < 0.001; figure 1*e*), indicating that the colour statistics of all three natural environments are biased in a blue–yellow direction. However, the three environments differed in the *extent* of the blue–yellow bias: log axis ratios differed significantly between locations (*F*(2,3740) = 146.4, *p* < 0.001). Post hoc Tukey tests revealed that log axis ratios were significantly different between each pair of environments (*p* < 0.001) and were largest in Quito, indicating that colours in Quito are most blue–yellow biased. A Bayesian ANOVA found a sensitive difference between locations (BF_10_ = 2.4 × 10^58^). Bayesian post hoc tests revealed that all the pairwise comparisons between locations were sensitive and different (BF_10_ > 40 939). The montages in figure 1*a* indicate that stronger blues in Quito than at the other two locations are likely to be contributing to Quito’s greater blue–yellow bias. The results of the one-way ANOVAs above inform the interpretation of the significant interaction. Axis ratios for discrimination thresholds for participants in Quito were significantly *smaller* (less blue–yellow biased) than for participants at the other two locations, while axis ratios for chromaticities in the environment in Quito were significantly *larger* (more blue–yellow biased) than for the other two locations. An additional analysis confirmed that differences between environments in log axis ratio were similar for light and dark pixels (see electronic supplementary material ‘Comparison of light and dark pixels’).

To investigate the potential impact of CVD on log axis ratios, we calculated the ratio of *L*/(*L* + *M*) to *S*/(*L* + *M*) thresholds for all participants. We identified six outliers in this threshold ratio (two from each group) who may potentially have CVD. However, CVD should not necessarily impact log axis ratios, as they are calculated in a colour space where the variances in thresholds along the *L*/(*L* + *M*) and *S*/(*L* + *M*) axes are independently normalized. We found, indeed, that the six outiers for the threshold ratio were not as a group outliers for log axis ratio, so there was no reason to exclude them from the main analysis (see electronic supplementary material, ‘Analysis of potential effect of CVD on blue–yellow bias’.)

As RGB cameras have a limited colour gamut relative to the gamut of the human visual system, RGB gamuts may constrain image statistics differently from the human colour gamut. We therefore also analysed several published sets of hyperspectral images that capture full visible spectra and found similar axis ratios for the hyperspectral image sets as for the image sets gathered using the head-mounted cameras (electronic supplementary material, figure S5). We then investigated the effect of the head-mounted cameras’ RGB gamuts on axis ratios by modelling the specific camera-acquired RGB values for the same hyperspectral images. We found that the constraints imposed by the cameras’ RGB gamuts did not lead to biased axis ratios for distributions of chromaticities in natural scenes captured in hyperspectral images (electronic supplementary material, figure S5).

### (b) Preferences for chromatic distributions and distributions of chromatic contrasts in natural scenes

Preferences for the differently coloured Mondrian patterns (figure 2*a*), as a function of the chromatic axis, are shown in figure 2*d,f*. On average, participants in Sussex preferred Mondrians with a greenish blue–orange hue axis (157.5–337.5°) and had the lowest preferences for a green–magenta (45–225°) hue axis. In contrast, the Chachi participants from Quito showed the opposite pattern and had the highest average preference for green–magenta and the lowest for orangish yellow–blue (135–315°). Chachi participants from Esmeraldas showed another pattern of results, where no Mondrian was preferred on average over any other. A repeated measures ANOVA with Mondrian hue angle (eight levels) as a within-subjects factor, location (three levels) as a between-subjects factor and preference as the dependent variable confirmed a significant hue angle × location interaction (*F*(14,483) = 5.32, *p* < 0.001, *η*_*p*_^2^ = 0.134; BF_10_ = 435.0), indicating that preference patterns differed across locations. Simple main effects for each location individually revealed that only for Sussex (*F*(7,126) = 4.84, *p* < 0.001, *η*_*p*_^2^ = 0.212; BF_10_ = 1691.0) and Quito participants (*F*(7,161) = 3.22, *p* = 0.003, *η*_*p*_^2^ = 0.123; BF_10_ = 26.2) was there a main effect of hue angle on preference, while for Esmeraldas participants, there was no significant effect (*F*(7,196) = 1.26, *p* = 0.271; BF_10_ = 0.076). A permutation analysis that established the null distribution of ranks for the preference ranking task (see the grey shaded areas in figure 2*d,f*) confirmed that there was a systematic influence of hue angle on preferences for the Quito and Sussex groups (see electronic supplementary material, ‘Preference, permutation analysis’).

In order to extract image statistics relevant to the Mondrian stimuli and the preference task, the chromaticity diagram was divided into 16 wedges corresponding to the axes along which the colours of the Mondrians were selected (i.e. 22.5° wedges centred on the poles of each stimulus axis shown in figure 2*a*). Each wedge was paired with its opponent wedge (e.g. 0° and 180°), and for each image, the saturations (eccentricity from the achromatic point in the scaled chromaticity diagram) of the pixels within each of the eight opponent wedges were averaged (figure 2*b*). This chromatic statistical analysis provided eight values per image captured, quantifying the mean chromatic contrast present in that image along each of the colour axes that defined the Mondrian stimuli in the preference task. The variation in the mean environmental chromatic contrast across the eight hue angles that defined the Mondrian stimuli interacted significantly with environment location, (*F*(14,25641) = 157.28, *p* < 0.001, *η*_*p*_^2^ = 0.079). One-way ANOVAs on the mean chromatic contrasts at each hue angle revealed differences between the environments for all hue angles (smallest *F* = 12.52, largest *p* < 0.001).

To address the central hypothesis that preferences are tuned to the chromatic statistics of local environments, we correlated mean preferences across the eight Mondrians with the set of eight corresponding mean saturations extracted from the image sets for each location. If preferences are tuned to the local environment, the correlation between preferences and image statistics should be highest for each group of participants when the image statistics are estimated from their local environment. Spearman’s rank correlations over the eight pairs of observations—the mean preference and corresponding mean saturation extracted from the image set—showed that there was a strong relationship between the preferences of Sussex and Quito participants and image statistics extracted from all locations (figure 2*c*). The strongest correlations were observed when mean preferences were correlated with the image statistics derived from the Esmeraldas image set (*ρ*(Sussex preferences/Esmeraldas environment) = 0.96, *p* < 0.001; *ρ*(Quito preferences/Esmeraldas environment) = −0.91, *p* = 0.005), indicating that there is no location-specific relationship between image statistics and preferences. Indeed, within each group, preferences were not predicted significantly better by the image statistics from one location than by the image statistics from any other location. The largest difference in correlation coefficients was between Sussex preferences and the Esmeraldas environment (*ρ* = 0.96) and between Sussex preference and the Sussex environment (*ρ* = 0.69), but this was not significant (Zou’s [36,37] 95% confidence interval for the difference between correlation coefficients included zero (−0.9893, 0.0314)). An additional analysis revealed similar patterns of results for light and dark image pixels (electronic supplementary material, ‘Comparison of light and dark pixels’).

We also analysed the same colour image statistics for hyperspectral images and for a model of the responses of the RGB sensors of the head-mounted cameras when exposed to the same hyperspectrally imaged scenes. We found similarly high correlations between preferences and colour statistics derived from hyperspectral image sets as for the image sets gathered using the head-mounted cameras. Correlations were also similarly high between preferences and the modelled head-mounted camera images of the hyperspectrally imaged scenes. This showed that the cameras’ RGB gamuts did not bias the colour statistics we extracted from the images acquired using the head-mounted cameras (see electronic supplementary material, ‘Hyperspectral image set and RGB Gamut analysis’).

## 4. Discussion

Our results reveal group differences in both colour discrimination and preferences. We also found significant differences in colour statistics between environments. We found, in accordance with earlier work, that both colour discrimination and preferences were broadly aligned with the colour statistics of natural scenes. However, the group differences in perception were not related to the differences in colour statistics between environments as would be expected under efficient coding frameworks. Instead, we found differences in colour discrimination that could be accounted for by perceptual learning and differences in preferences that could be accounted for by an interaction between culture and perceptual fluency.

To relate colour discrimination and the colour statistics of natural scenes, we extracted log axis ratios describing the blue–yellow bias present in images gathered at three locations. We found significant differences in log axis ratio between locations. Quito images had the largest log axis ratios, indicating that colour variance is most dominant along the blue–yellow axis at this location. Esmeraldas images had the smallest log axis ratios, and Sussex images had intermediate log axis ratios. Perceptually, we found subtle differences in the log axis ratios for colour discrimination ellipses between locations, which were statistically significant using parametric statistics but generated insensitive Bayes factors. Participants in Quito had significantly smaller log axis ratios than those in Sussex, indicating relatively better saturation discrimination along the blue–yellow axis, and there was a trend for participants in Quito to also have smaller log axis ratios than participants in the Esmeraldas. Under the efficient coding framework, the relatively greater chromatic variation along the blue–yellow axis in Quito natural scenes should produce *larger* log axis ratios perceptually. In a range-accuracy trade-off, the greater range of colour contrasts along the blue–yellow axis in the natural environment should be represented with lower fidelity by the visual system and lead to relatively higher discrimination thresholds. Instead, our results implied the opposite: participants in Quito who are exposed to natural scenes more biased along the blue–yellow axis have *less* biased colour discrimination ellipses. Our finding that the discrimination ellipses for all three groups are aligned with the blue–yellow bias measured in all three environments is broadly compatible with the efficient coding framework: greater blue–yellow contrasts in natural scenes are coupled with reduced sensitivity to blues and yellows. However, the pattern of group differences in the amount of blue–yellow bias in environment and colour discrimination provides no evidence for within-lifetime calibration to environmental colour statistics under the efficient coding framework. Our discrimination results provide tentative evidence there may be a within-lifetime effect in the opposite direction to that predicted by efficient coding that is nonetheless aligned with scene statistics: people living in the most blue–yellow biased environment showed the least blue–yellow bias in their colour discrimination. Such an opposite effect could be understood in the alternative frameworks of perceptual learning or perceptual expertise with more common colours [38], where greater exposure to blues and yellows *increases* perceptual sensitivity to blues and yellows, reducing the blue–yellow bias. Taken together, our findings are compatible with the idea that over evolution there is calibration of colour sensitivity to the ranges of colours present in natural scenes under the efficient coding framework, but that any within-lifetime effects are driven by perceptual learning.

In addition to the group differences in the amount of blue–yellow bias in the discrimination ellipses, there were also group differences in the overall sizes of the discrimination ellipses that suggest overall lower discrimination thresholds for Sussex than Quito or Esmeraldas participants. We find no evidence for an environmental account for such differences in overall colour discrimination performance, in that the sizes of the colour gamuts of visual diets across groups are approximately equal. Our results may indicate group differences in visual sensitivity to colour, for example, via differences in macular pigment or lens density. However, these particular factors on which individuals vary are specific to particular colour axes and would not be expected to alter discrimination performance uniformly for all hue angles. Alternatively, these group differences in overall discrimination performance could arise from differences in responses to non-visual task demands such as motivation or familiarity with iPads or scientific experiments. We therefore urge caution in interpreting the observed group differences in the sizes of discrimination ellipses.

To examine the relationship between preferences for chromatic distributions and natural scene statistics, we extracted the mean saturation in natural scenes along each of the hue axes of our Mondrian stimuli. For all locations, the functions describing how mean saturation varies with the hue axis have a peak at the blue–yellow axis and a minimum along the orthogonal green–magenta axis. Though these curves are highly correlated between locations, they do differ significantly. For participants in the Esmeraldas, we did not find significant differences in preferences between colour axes. Our results for the Esmeraldas participants are based on a different method than those of the other two groups (electronic supplementary material, ‘Preference rating method’): participants in the Esmeraldas gave preference ratings for the Mondrian stimuli, while Quito and Sussex participants ranked sets of Mondrian stimuli for preference. However, we found that the rating method used in the Esmeraldas could reveal expected preferences for familiar objects (see electronic supplementary material, ‘Preference rating method’), and for Sussex participants, there was a similar preference curve with a peak in the same location for the two different methods, though the rating method appears to be less sensitive to variation in preferences than the ranking method (see electronic supplementary material, ‘Comparison of preferences from rating and ranking methods for Sussex participants’). Our results suggest that participants in the Esmeraldas simply do not have strong preferences for one chromatic distribution over another. Participants in Quito and Sussex each show preference curves that are relatable to the variation in saturation with hue axis in natural scenes, but the preference curves have opposite polarities. We found no statistical evidence that the group mean preference curves are more aligned with the local environment than with the environments of other groups. The preference curves of Quito and Sussex participants were both significantly correlated, though with opposite signs, with curves describing scene statistics at all three locations. However, the correlation coefficients between preference and scene statistic curves were larger, although not significantly so, for Esmeraldas images than for Quito or Sussex images. The preference curve for Sussex participants was aligned with the colour statistics of natural scenes, with the strongest preferences near the blue–yellow axis, which had the highest mean saturation in natural scenes. For Quito participants, Mondrians along the blue–yellow axis were least preferred. Quito participants seem to prefer Mondrians that differ most from the statistics of natural scenes.

There are two different aspects of the Mondrian stimuli that could drive preference ratings. It may be that particular pairs of *hues* are more preferred than others. Alternatively, participants may prefer Mondrians that have either low or high perceived chromatic *contrast* (the differences in perceived saturation between the colours within a Mondrian). Preferences for particular hue axes might be driven by the probability of encountering such hues in natural scenes, or by the positive valence of objects, scenes or concepts associated with distributions of colour along particular hue axes (analogously to Palmer & Schloss’s ecological valence hypothesis [39]), or by differences in colour harmony for hue pairs between hue axes [40].

Since we defined our Mondrians along an isosaturated circle in the MacLeod–Boynton [29] chromaticity diagram, Mondrians along the blue–yellow axis would appear to be of lower chromatic contrast than those along the orthogonal green–magenta axis (due to the relatively poorer colour sensitivity along the blue–yellow axis we observe in colour discrimination ellipses). Under the perceptual fluency hypothesis [41], people may experience lower contrast patterns as more visually comfortable than higher contrast patterns if they are encoded more efficiently by the visual system. Low visual discomfort may lead to high aesthetic preferences [7], or alternatively to low aesthetic preferences if people prefer images they find more challenging. Efficient coding could potentially therefore explain the patterns of results we see for both Sussex and Quito groups, but our findings may suggest that perceptually fluent stimuli are not always preferred. In favour of perceived chromatic contrast as a driver of preferences for our Quito participants, preferences for single colour patches in Quito participants are driven by saturation and not by hue (as in some other cultures [42]; see electronic supplementary material, ‘Preferences for single colours in Quito participants’).

While the groups are likely to differ in their colour associations and symbolism due to cultural variation in their interactions with colour [43] (see electronic supplementary material, ‘Information about the Chachi population’), it is difficult to see how cultural variation in associations with single hues could account for the shapes of the preference curves being highly similar for Sussex and Quito groups but of reverse polarity. However, the opposite patterns of results for Quito and Sussex participants do suggest that preferences for colour distributions (whatever their determinants) are culturally mediated. One possibility is that there is cultural variability in preferences for perceived chromatic contrast, with some cultures preferring more sensory stimulation in certain contexts than others. Further experiments could arbitrate between hue preferences or contrast preferences as determinants of Mondrian preference curves.

The efficient coding framework has been proposed as an explanation for the blue–yellow bias observed in both colour discrimination ellipses [4,22] and in patterns of preference for distributions of colour [7]. Though efficient coding may still provide an explanation for blue–yellow biases through evolution, our colour discrimination results do not provide support for the idea that there is calibration of perception via efficient coding to local environments within a lifetime. Rather, our discrimination results favour the idea that group differences may arise via perceptual learning through familiarity with more common colours. Critically, our preference results suggest that any account of preference via efficient coding must be culturally relative, as the relationships between preference and contrast are in opposing directions across groups. Here we have investigated whether group differences in perception can be accounted for by group differences in visual diets. Future research could use our approach to investigate whether within-group individual differences are influenced by calibration to individual observer visual diets. This would require a test–retest design for the perceptual tests to capture stable individual differences, and matched data within observers for perceptual and image statistic measures. Future research could also investigate within-individual variation across seasons. As the colour statistics of environments can exhibit seasonal changes [44], we might expect to find corresponding changes in colour perception over seasons if it is calibrated within lifetimes to the colour statistics of visual diets [25].

Our cross-environmental approach to investigating the calibration of discrimination and preferences to natural scene statistics could be applied to other types of statistical regularities, such as those of spatial structure [8]. For research on perception as a relatively ‘low-level’ cognitive process, there is often a universalist assumption that perceptual attributes and processes are hardwired and common to all. However, we know that experience matters for perception [9] and aesthetics [45], which means we need to measure perception in populations other than ‘WEIRD’ ones to characterize human perception more accurately [46]. Psychophysical studies requiring carefully controlled visual stimuli are obviously challenging to conduct outside the lab, but new hardware is making this more achievable. Our calibrated iPad displays viewed inside blackout tents are one solution to presenting controlled stimuli in the field. To investigate the effect of experience on perception and aesthetics, it is important to accurately capture the lived visual diet. Our method of using calibrated head-mounted cameras to capture egocentric visual inputs along with modelling low-level visual responses could be applied in many research contexts, including visual development, further cross-cultural work, or animal work. These tools will therefore be useful to further investigate the extent to which aspects of perception are efficiently tuned to visual diets and the extent to which perception differs between people living in different visual environments around the world.

## Data Availability

The data, code and README files are available at https://osf.io/hv4n6. The head-mounted camera images of people going about their daily lives cannot be shared for privacy and ethical reasons, but derived image statistics are shared along with the scripts to produce them. Supplementary material is available online [47].
